# Supplementary figures and images for: GLP-1 Notch—LAG-1 CSL control of the germline stem cell fate is mediated by transcriptional targets lst-1 and sygl-1
Source: PLoS Genet. 2020 Mar 20;16(3):e1008650. doi: 10.1371/journal.pgen.1008650 (PMC7153901; doi:10.1371/journal.pgen.1008650)

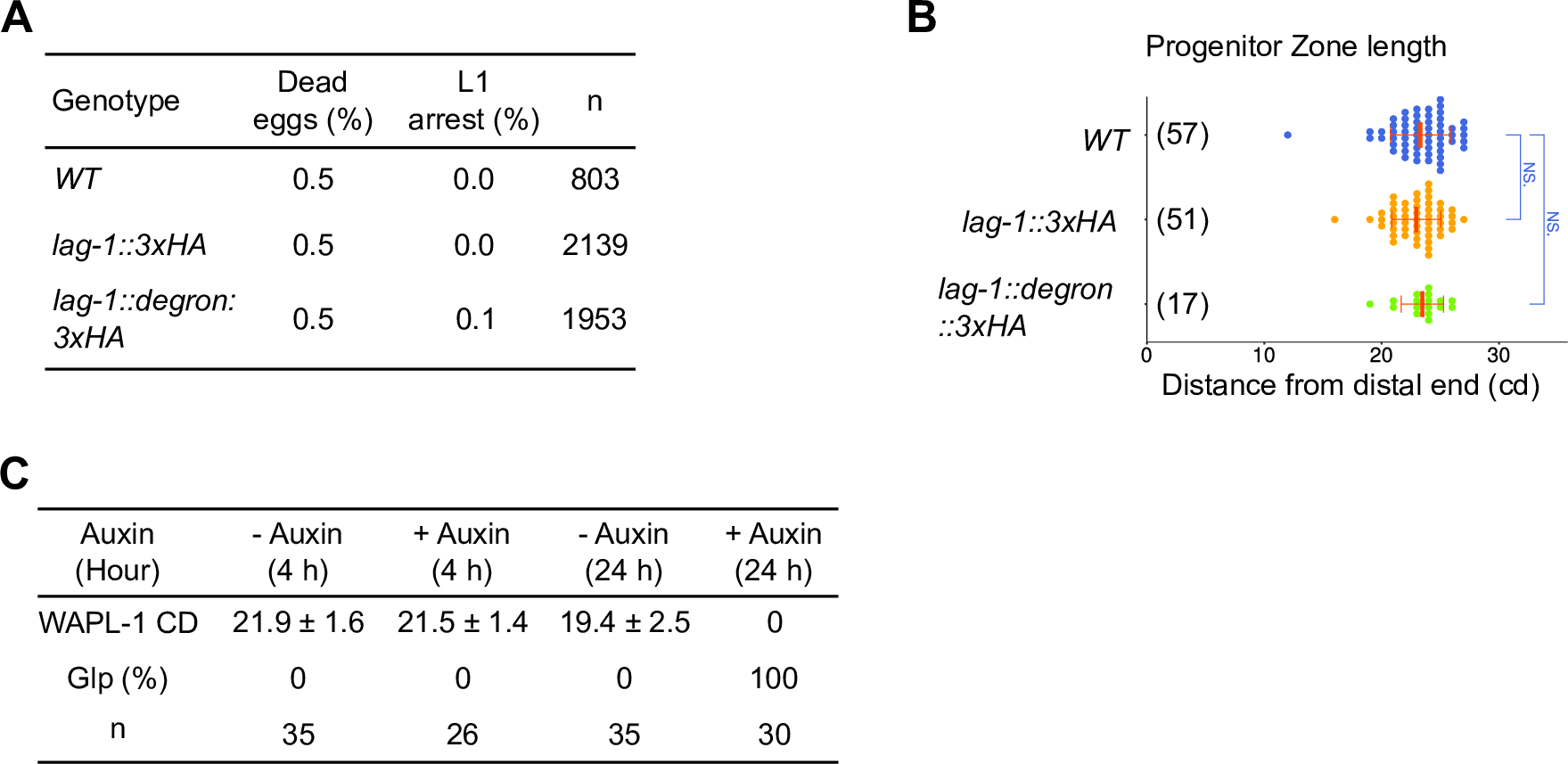

Supplement: S1 Fig — (A & B) The dead eggs and L1 arrest frequency (A) and progenitor zone length (B) in WT, lag-1(oz530[lag-1::3xHA]) and lag-1(oz536oz537[lag-1::degron::3xHA]). (B) Graph showing progenitor zone length, in cell diameters, between the distal tip of the germline and the row of cells at proximal end of the continuous zone of WAPL-1 staining for L4 hermaphrodites of indicated genotype. Data are plotted as horizontal dot plots with each dot representing length in cell diameter to zone end for one gonad. Numbers in bracket shows the sample size. Thick vertical lines represent mean and horizontal lines represent mean ± SD. P-value ≤ 0.01 (*); ≤ 0.001 (**); ≤ 0.0001 (***); > 0.01 non-significant (NS.). (C) Progenitor zone length and premature meiotic entry of progenitor zone cells (Glp) phenotype after L4 stage animals were treated with or without auxin for either 4 hrs or 24 hrs (in Fig 2D & 2E). The genotype for auxin treatment was lag-1(oz536oz537[lag-1::degron::3xHA]); ieSi64[gld-1p::TIR1::mRuby::gld-1 3'UTR]. The lag-1::3xHA and lag-1::degron::3xHA strains appears phenotypically wild type; we did not observe phenotypes associated with lag-1 loss of function [11,17], including loss of glp-1—embryonic lethality or a smaller progenitor zone due to stem cells undergoing spatially premature meiotic entry, loss of lin-12—egg-laying/vulva defects, or loss of both lin-12 and glp-1—Lag larval arrest. (TIF) [file pgen.1008650.s001.tif]

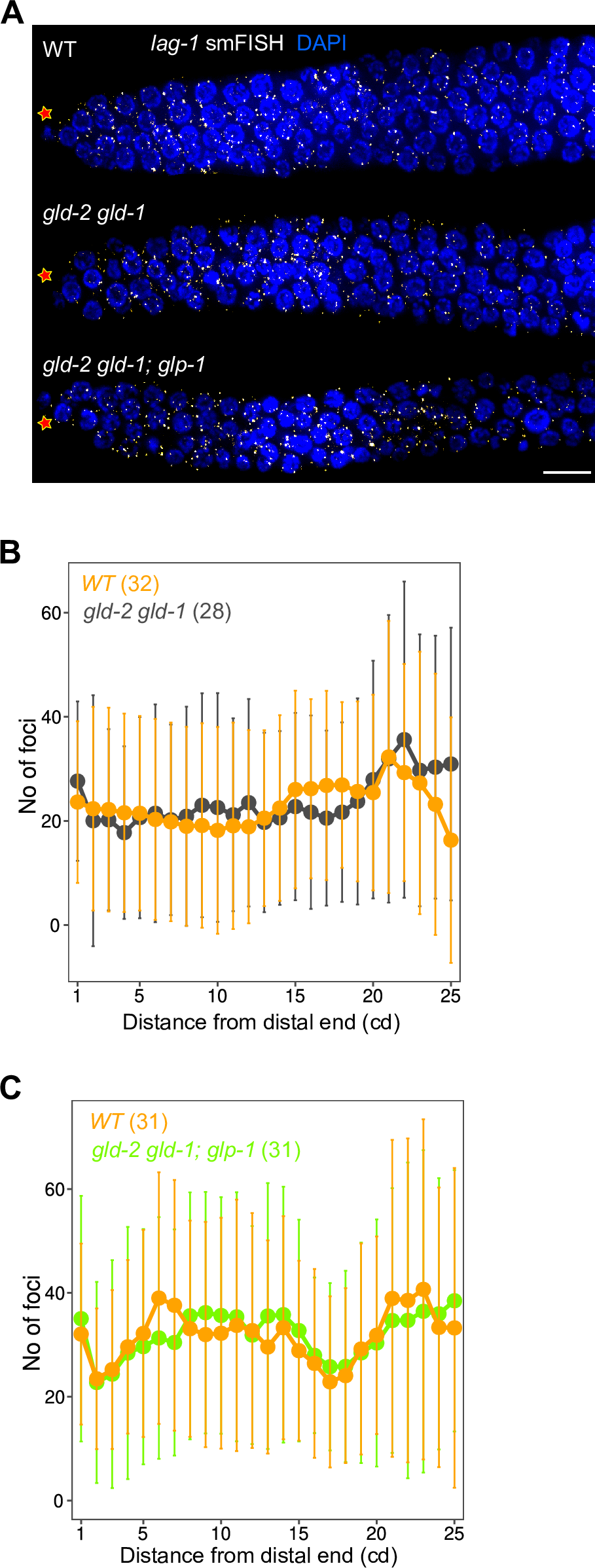

Supplement: S2 Fig — (A) Z projection of one nucleus thick (see Materials and Methods) distal germlines of L4 hermaphrodites, probed for LAG-1 transcripts using smFISH (white) and DAPI (blue). Star, distal end; Scale bar, 10 μm. (B & C) Density plot of lag-1 mRNA foci for indicated genotype. Numbers in bracket shows the sample size. Additionally, there was no difference in lag-1 mRNA level from GLP-1 ON versus GLP-1 OFF transcriptomics analysis. (TIF) [file pgen.1008650.s002.tif]

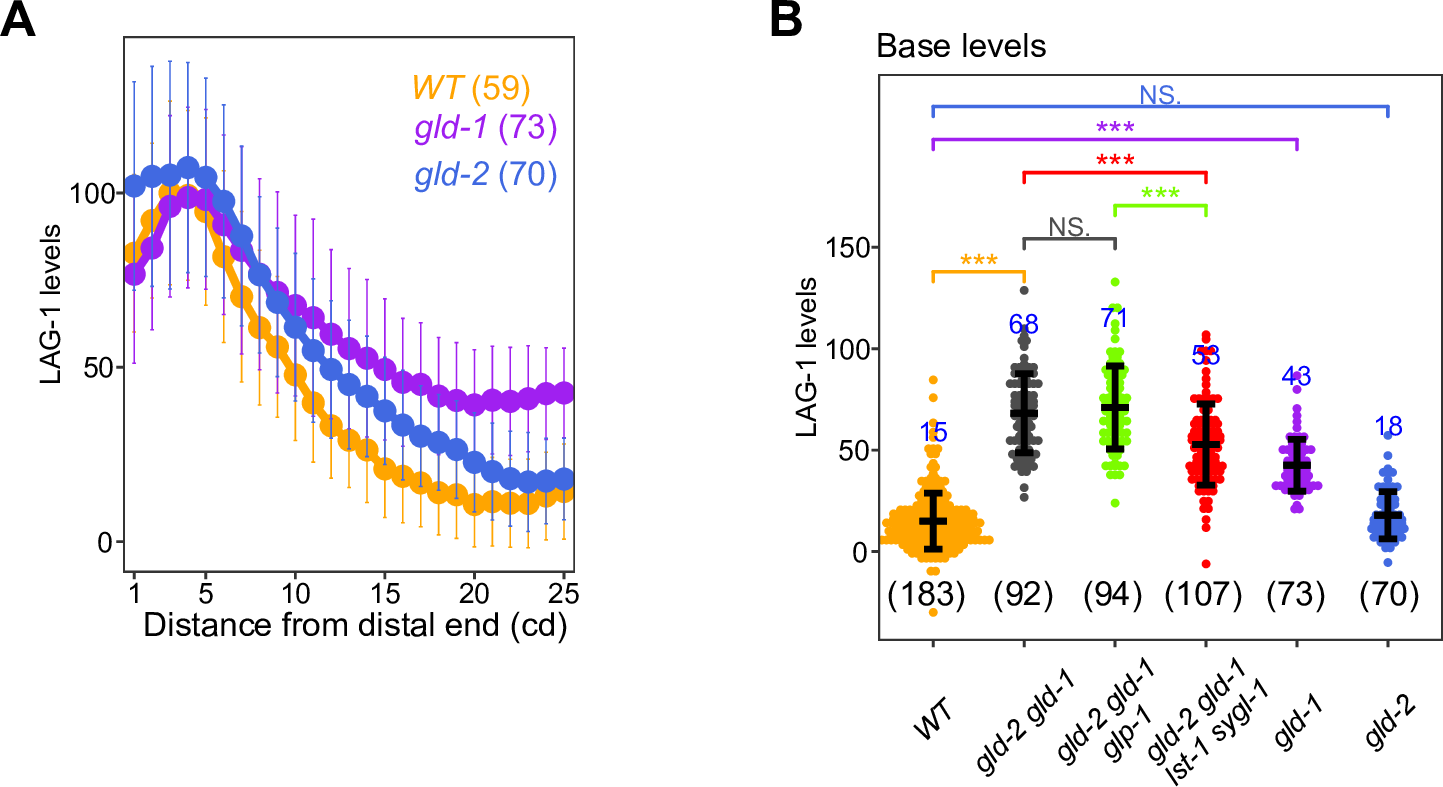

Supplement: S3 Fig — (A—B) Plot of LAG-1 levels (A) and comparison of LAG-1 base (B) for indicated genotype. lag-1(oz530[lag-1::3xHA]) was used for quantitation; See S1 Table for the complete genotypes. Numbers indicate mean values of LAG-1 level for each genotype and numbers in bracket shows the sample size. Dots, mean (A) or data points (B); Error bars, mean ± SD. P-value ≤ 0.01 (*); ≤ 0.001 (**); ≤ 0.0001 (***); > 0.01 non-significant (NS.). (TIF) [file pgen.1008650.s003.tif]

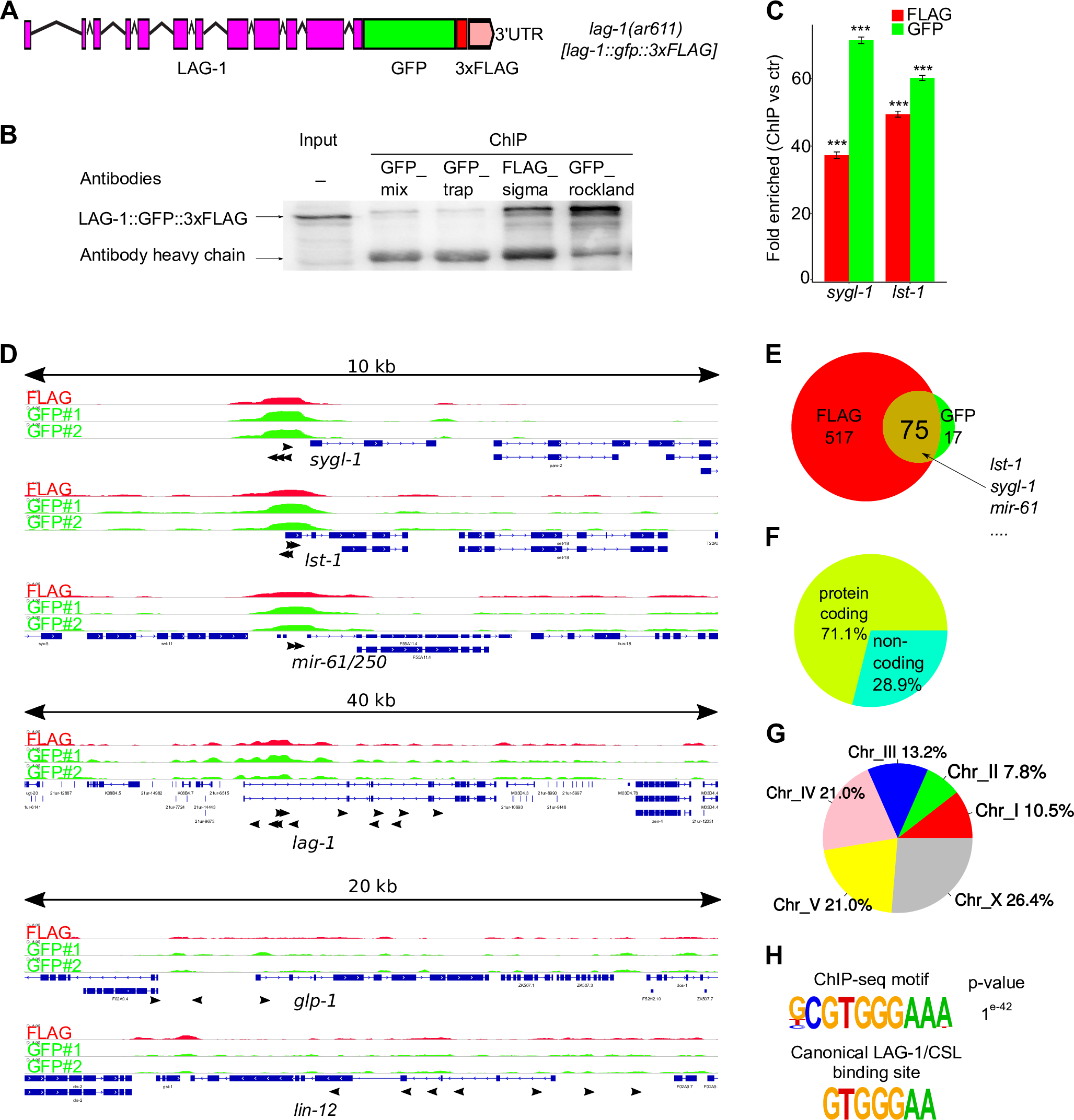

Supplement: S4 Fig — (A) Diagram of lag-1 allele at endogenous locus, lag-1(ar611[lag-1::gfp::3xFLAG]) (gift from Iva Greenwald), used for whole worm ChIP-seq analysis. (B) Different antibodies were tested to determine if they are competent for ChIP. Same amount of extracts were used for each ChIP experiment, followed by western blot analysis with FLAG antibody. Bottom band detects the heavy chain of antibodies. FLAG antibody from Sigma and GFP antibody from Rockland were selected for the analysis below. (C) ChIP-qPCR analysis for sygl-1 and lst-1 promoter regions bound by LAG-1. A non-peak region in the xol-1 promoter was used as a negative control (ctr) and set as 1. *** for p<0.0001. Error bars, mean ± SD. (D) Genome browser tracks showing 10 kb genomic region for sygl-1, lst-1 and mir-61/250, 40 kb genomic region for lag-1, and 20 kb genomic region for glp-1 and lin-12 after ChIP-seq. Raw reads were normalized to control, and signal intensity were presented as log2 fold change. Black arrow heads, canonical LAG-1/CSL binding motif GTGGGAA [16,18,19]. (E) Venn diagram showing the overlapping genes identified through FLAG antibody and GFP antibody ChIP-seq analysis. Both data lists were filtered for more than 2-fold change of signal (ChIP/control) with a moderate False Discovery Rate (FDR<0.05). (F & G) Protein coding vs. non-coding distribution (F) and chromosome position (G) of 75 genes from E. The overly represented motif discovered by HOMER suite with the ChIP-seq data (top) and the canonical LAG-1/CSL binding sequence [16,18,19](bottom). See S3 Table. (H) The over-represented motif discovered by HOMER suite with whole animal ChIP-seq data (top) and the reported canonical LAG-1/CSL binding motif (bottom) [16,18,19]. (TIF) [file pgen.1008650.s004.tif]

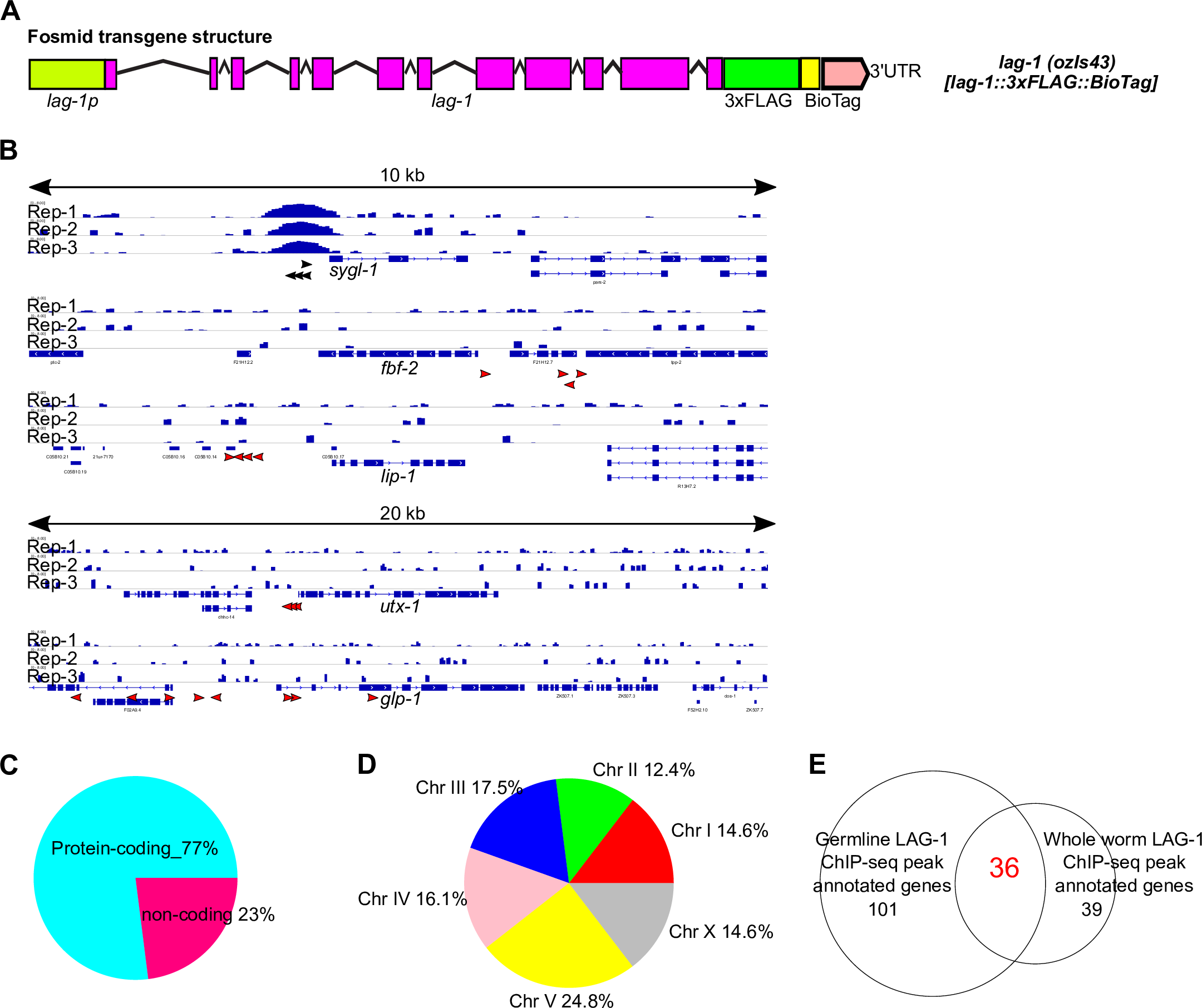

Supplement: S5 Fig — (A) Diagram of lag-1(ozIs43[lag-1p::lag-1::3xFLAG::BioTag::lag-1 3’UTR]) fosmid transgene. 3xFLAG and BioTag sequence were inserted into a fosmid that contains native regulatory sequence for lag-1 gene. lag-1(ozIs43) was able to rescue lag-1 deletion allele tm3052. (B) Genome browser tracks showing sygl-1 and four other putative germline GLP-1/LAG-1 transcriptional targets from literature: 10 kb genomic region for sygl-1 (this study, [20]), fbf-2 [30] and lip-1 [31], 20 kb genomic region for utx-1 [32] and glp-1 [16]. Raw reads were normalized to control, and the signal intensity were presented as log2 fold change. Black arrow heads, canonical LAG-1/CSL binding motif GTGGGAA [16,18,19]. Red arrow heads, LAG-1 binding site (LBS) from original references where LAG-1 was suggested to bind. (C & D) Protein coding/ non-coding distribution (C) and chromosome position (D) of 137 genes from Fig 4D. (E) Venn diagram showing the overlapping genes from germline and whole worm ChIP-seq analysis of LAG-1. See S4 Table. (TIF) [file pgen.1008650.s005.tif]

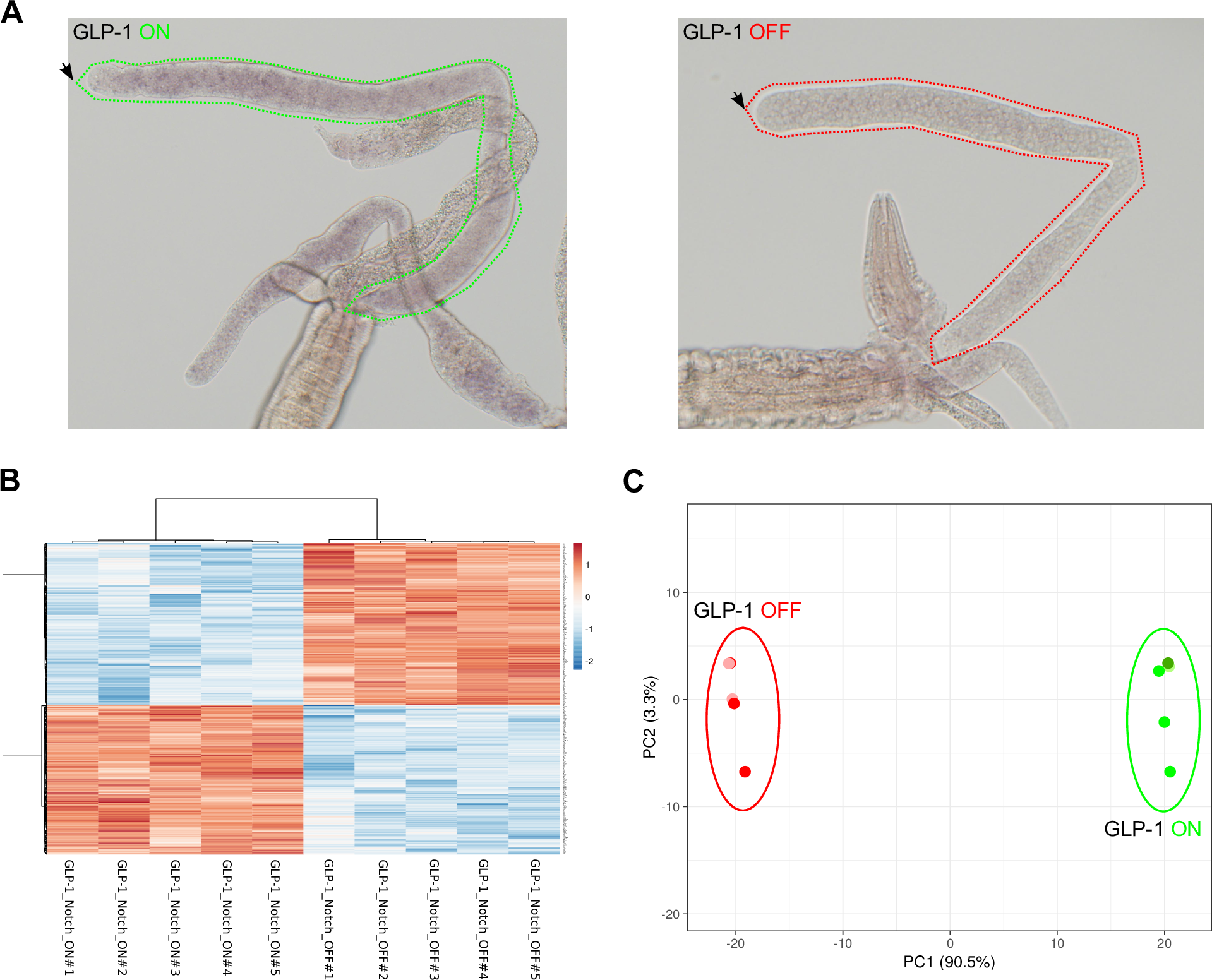

Supplement: S6 Fig — (A) In situ hybridization used to determine lst-1 mRNA expression in young adult animals. The genotypes are, GLP-1 ON: gld-2(q497) gld-1(q485); glp-1(ar202) and GLP-1 OFF: gld-2(q497) gld-1(q485); glp-1(q175). Dotted lines outline the boundary of the gonad. Black arrow indicates the distal tip of the gonad. (B & C) Heatmap (B) and principal component analysis (PCA) (C) were generated using the top 500 genes with the most significant p-value from differential gene expression analysis. (TIF) [file pgen.1008650.s006.tif]

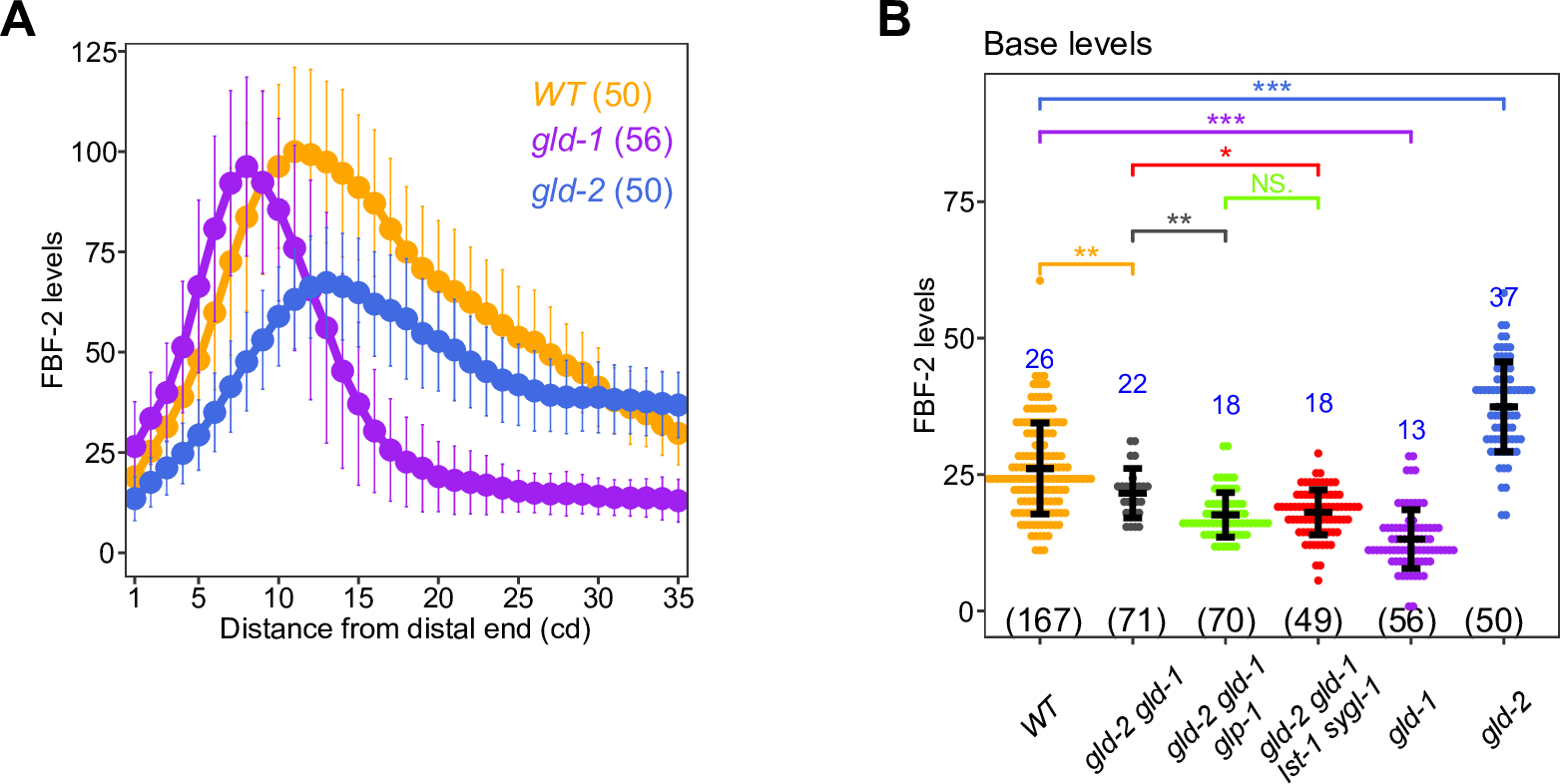

Supplement: S7 Fig — (A—B) Plot of FBF-2 levels (A) and comparison for FBF-2 base (B) for indicated genotype. fbf-2(q932[3xV5::fbf-2]) is used for quantitation. See S1 Table for the complete genotypes. Numbers indicate mean values of FBF-2 level for each genotype and numbers in bracket shows the sample size. Dots, mean (A) or data points (B); Error bars, mean ± SD. P-value ≤ 0.01 (*); ≤ 0.001 (**); ≤ 0.0001 (***); > 0.01 non-significant (NS.). (TIF) [file pgen.1008650.s007.tif]

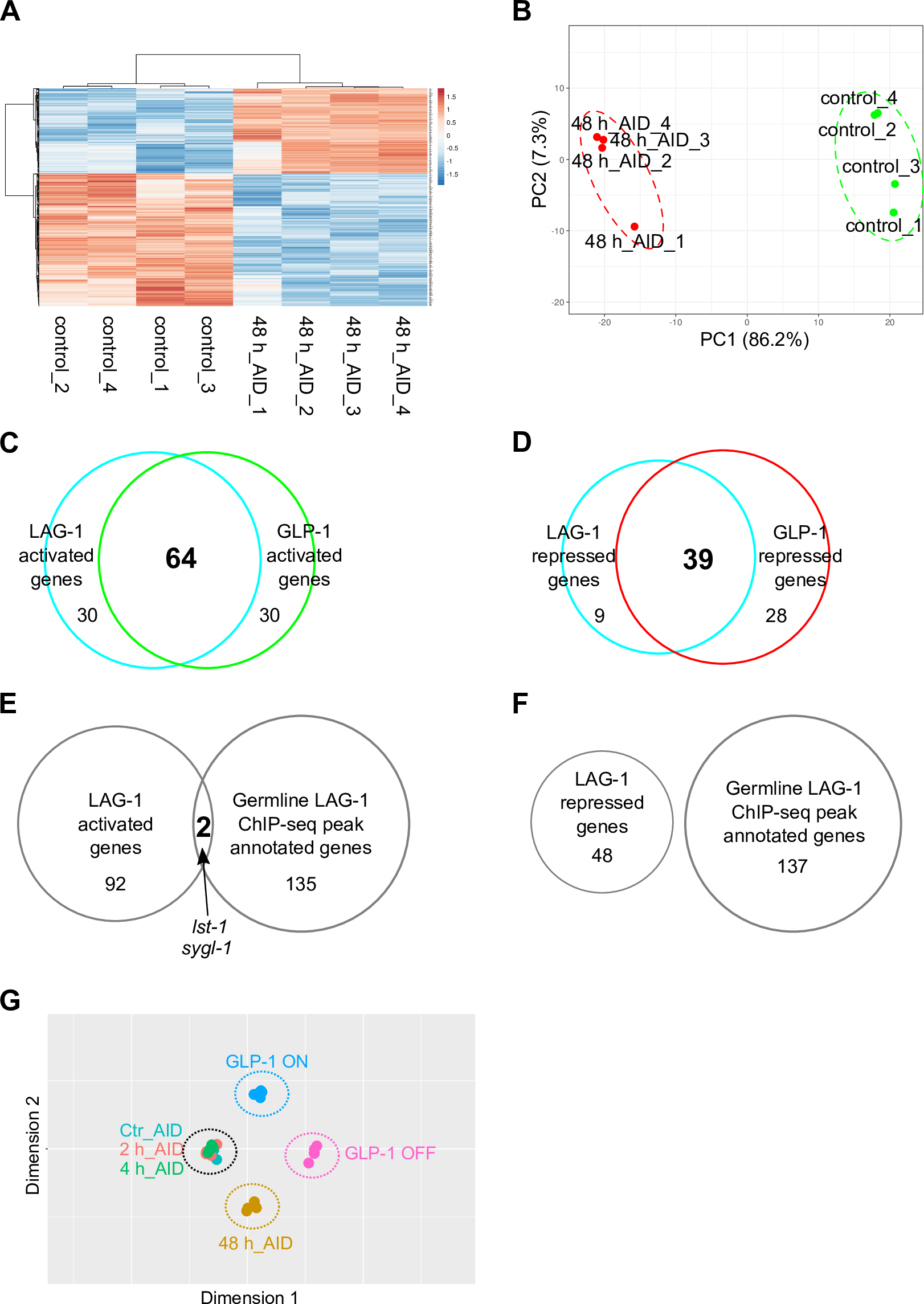

Supplement: S8 Fig — (A & B) Heatmap (A) and principal component analysis (PCA) (B) for top 500 genes with most significant p-values, with the differential gene expression analysis done between animals treated with or without auxin for 48 hours. (C & D) The differentially-expressed genes upon auxin treatment for 48 hours were compared to the differentially-expressed genes in GLP-1 ON vs. OFF to identify the overlapping genes activated (C) or repressed (D) by both LAG-1 and GLP-1. (E & F) The differentially-expressed genes upon auxin treatment for 48 hours were compared to putative LAG-1 targets through LAG-1 germline ChIP-seq analysis to determine the LAG-1 transcriptional targets (E) and if LAG-1 can repress gene expression (F). (G) Multiple dimensional scaling analyses showing the similarities of the RNA-seq samples conducted in this study. Five biological replicates each were conducted for GLP-1 ON (in blues circle) and GLP-1 OFF (in pink circle). Four biological replicates were conducted for time course RNA-seq analysis following LAG-1 degradation by auxin treatment. The 48-hour auxin treated samples were grouped in yellow circle. For the shorter period of treatment, 2 hours and 4 hours, the transcriptomic profile are similar to the untreated samples and were grouped in the black circle. See S6 and S7 Tables. (TIF) [file pgen.1008650.s008.tif]

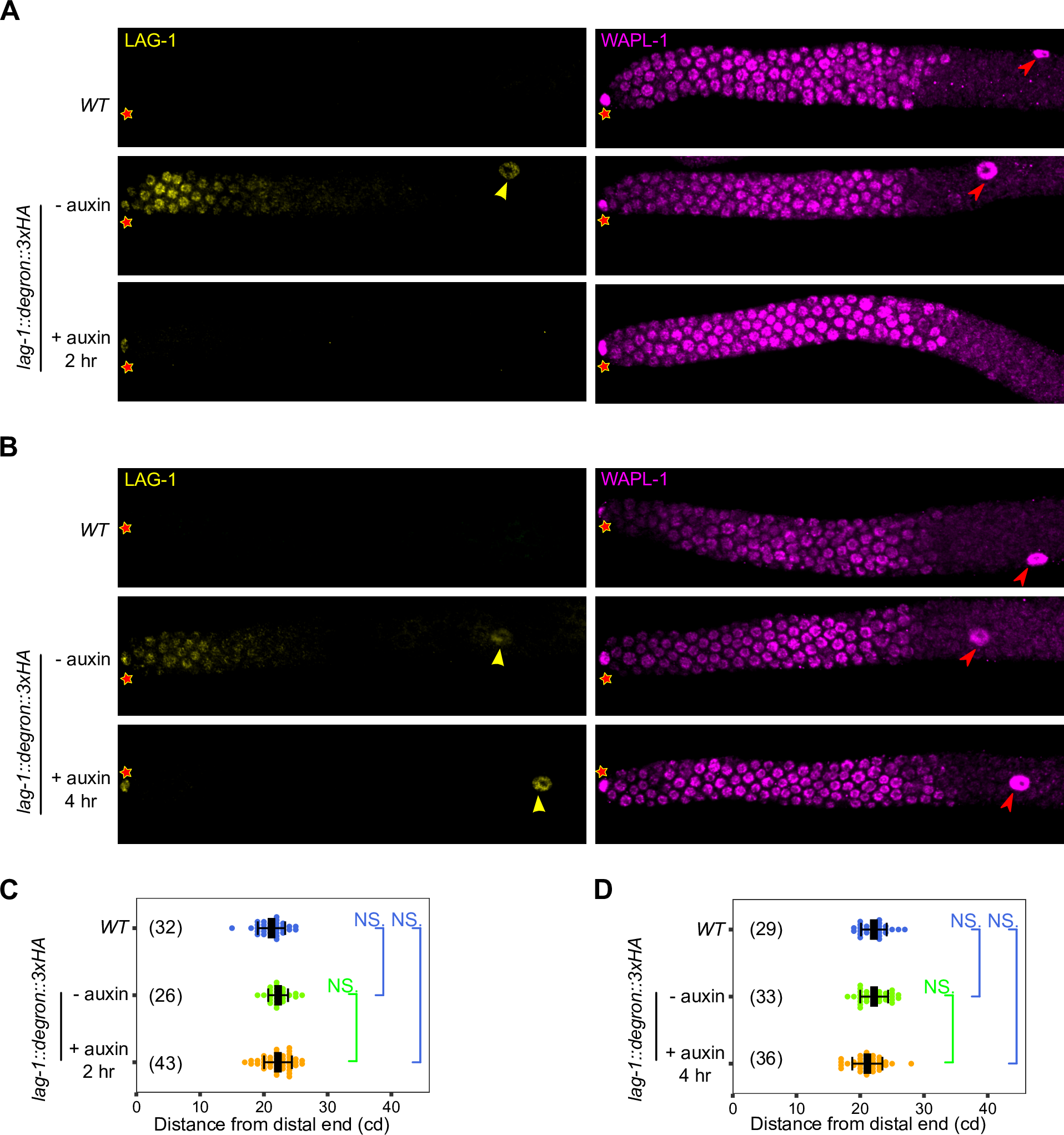

Supplement: S9 Fig — (A & B) Representative images of anti-HA-stained (LAG-1, yellow, left panels) and WAPL-1-stained (pink, right panels) after L4 stage animals were treated with or without auxin for 2 hours (A) or 4 hours (B) at 25°C. Star, distal end of germline. Yellow arrowheads, LAG-1 accumulation in the sheath cells, red arrowheads, WAPL-1 accumulation in sheath cells. (C & D) Graph showing distance, in cell diameters, between the distal end of the germline and the row of cells at proximal end of the continuous zone of WAPL-1 staining for auxin-treated hermaphrodites. Data are plotted as horizontal dot plots with each dot representing length in cell diameter to zone end for one gonad. Numbers in bracket shows the sample size. Thick vertical lines represent mean and horizontal lines represent mean ± SD. P-value ≤ 0.01 (*); ≤ 0.001 (**); ≤ 0.0001 (***); > 0.01 non-significant (NS.). (TIF) [file pgen.1008650.s009.tif]

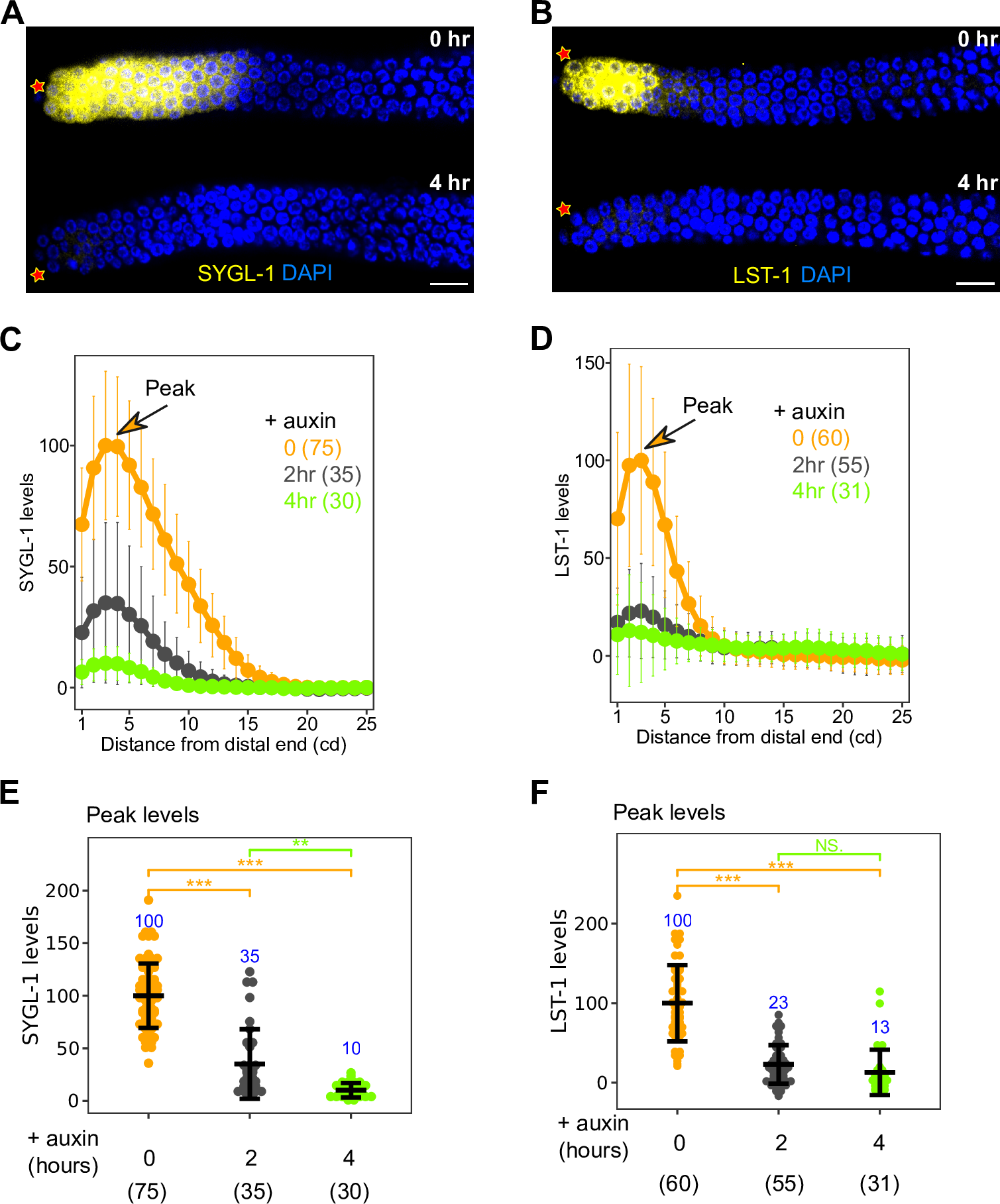

Supplement: S10 Fig — (A & B) Representative images of SYGL-1 (A) and LST-1 (B) accumulation after animals were treated with or without auxin for 4 hours. The genotype for auxin treatment are sygl-1(q983[3xOLLAS::sygl-1]); lag-1(oz536oz537[lag-1::degron::3xHA]); ieSi64[gld-1p::TIR1::mRuby::gld-1 3'UTR] (for quantitation of SYGL-1 levels) and lst-1(q1003[lst-1::3xOLLAS]); lag-1(oz536oz537[lag-1::degron::3xHA]); ieSi64[gld-1p::TIR1::mRuby::gld-1 3'UTR] (for quantitation of LST-1 levels). Note that the images, taken at identical exposure time, were processed identically to make residual protein visible in the auxin treated germlines, this resulted in slightly saturated signal in the untreated germlines. (C & D) Plots of SYGL-1 (C) and LST-1 (D) levels in auxin-treated germlines. Numbers in bracket shows the sample size. Dots, mean. Error bars, mean ± SD. (E & F) Comparison of SYGL-1 (E) and LST-1 (F) peak levels (see C & D) in auxin-treated germlines. Numbers indicate mean values for each genotype and numbers in bracket shows the sample size. Dots, data points. Error bars, mean ± SD. P-value ≤ 0.01 (*); ≤ 0.001 (**); ≤ 0.0001 (***); > 0.01 non-significant (NS.). (TIF) [file pgen.1008650.s010.tif]
